# Estimating the costs of induced abortion in Uganda: A model-based analysis

**DOI:** 10.1186/1471-2458-11-904

**Published:** 2011-12-06

**Authors:** Joseph B Babigumira, Andy Stergachis, David L Veenstra, Jacqueline S Gardner, Joseph Ngonzi, Peter Mukasa-Kivunike, Louis P Garrison

**Affiliations:** 1 Global Medicines Program, Department of Global Health, School of Public Health, University of Washington, Seattle, WA, USA; 2 Pharmaceutical Outcomes Research and Policy Program, School of Pharmacy, University of Washington, Seattle, WA, USA; 3 Department of Epidemiology, School of Public Health, University of Washington, Seattle, WA, USA; 4 Department of Obstetrics and Gynecology, Mbarara University of Science and Technology, Mbarara, Uganda; 5 Engender Health Fistula Care Project, Kampala, Uganda

## Abstract

**Background:**

The demand for induced abortions in Uganda is high despite legal and moral proscriptions. Abortion seekers usually go to illegal, hidden clinics where procedures are performed in unhygienic environments by under-trained practitioners. These abortions, which are usually unsafe, lead to a high rate of severe complications and use of substantial, scarce healthcare resources. This study was performed to estimate the costs associated with induced abortions in Uganda.

**Methods:**

A decision tree was developed to represent the consequences of induced abortion and estimate the costs of an average case. Data were obtained from a primary chart abstraction study, an on-going prospective study, and the published literature. Societal costs, direct medical costs, direct non-medical costs, indirect (productivity) costs, costs to patients, and costs to the government were estimated. Monte Carlo simulation was used to account for uncertainty.

**Results:**

The average societal cost per induced abortion (95% credibility range) was $177 ($140-$223). This is equivalent to $64 million in annual national costs. Of this, the average direct medical cost was $65 ($49-86) and the average direct non-medical cost was $19 ($16-$23). The average indirect cost was $92 ($57-$139). Patients incurred $62 ($46-$83) on average while government incurred $14 ($10-$20) on average.

**Conclusion:**

Induced abortions are associated with substantial costs in Uganda and patients incur the bulk of the healthcare costs. This reinforces the case made by other researchers--that efforts by the government to reduce unsafe abortions by increasing contraceptive coverage or providing safe, legal abortions are critical.

## Background

Induced abortion is illegal in Uganda except to save the life of a pregnant mother and to preserve her physical and mental health [1]. Induced abortion is also the subject of substantial social stigma. The Catholic Church, the largest single religion in Uganda to which 42% of the population belong [2], strictly prohibits it [3], and the rapidly-growing evangelical movement condemns it [4]. Anti-abortion stigma has even been reported among the highly-educated [5] and health workers [6].

Yet the demand for induced abortion remains high, probably because of a high number of unintended pregnancies, at least 700,000 annually [6], which are a consequence of the low access to modern contraceptives among women who want to avoid pregnancies (31%) [7] and of other social factors such as poverty, illness, already having too many children, or abusive relationships [8-11]. Of the unintended pregnancies, almost 4 in 10 (38%) result in abortion and the rest continue and result in unintended births [6].

Women who decide to abort often resort to untrained and usually unskilled practitioners who practice in illegal and hidden clinics and often provide unsafe abortion procedures that result in a high rate of complications and sometimes death. Unsafe abortions are estimated to be the cause of 21% of all maternal deaths in Uganda [6] compared to about 13% of all maternal deaths globally [12] and are a major reason why the country has one of the highest levels of maternal mortality in the world [6,13,14]. Therefore the problem of unsafe abortion, while not unique to Uganda, is of special significance in this country. In 2003, there were an estimated 297,000 induced abortions performed that resulted in 85,000 complications treated in the health care system and 1,200 maternal deaths [15]. In 2009, the estimated number of induced abortions in Uganda was 362,000 [16] suggesting an upward trend.

Illegally-performed unsafe abortions in Uganda pose a large health risk for women because of inadequate skills of the providers, unsanitary environments, and hazardous techniques [17] which increase the rate of immediate complications such as severe bleeding, abdominal and genital injury, or death. If women survive the procedure, they may develop other complications--most commonly hemorrhage, sepsis, and genital perforation [18,19]. Such severe complications need complex tertiary care which is only available at referral public hospitals with the capacity to perform extensive surgical operations, blood transfusions, and intensive care. Patients with these complications tend to have long hospital stays with 57% staying for more than 13 days [19]. This results in consumption of large amounts of healthcare resources such as personnel, theatre space, medications, and hospital beds [20]. Some of the women who survive their hospital stay also suffer long-term complications such as pelvic infection, ectopic pregnancy, vesico-vaginal fistulae, urinary incontinence, utero-vaginal prolapse, infertility, and many mental health problems [21-24]. These complications also usually require specialist care and are associated with increased health resource utilization. In a country where total per capital health expenditure is only $44 [25], costs attributable to induced (usually unsafe) abortion may represent a substantial diversion of public healthcare resources from other disease areas which, if saved, could be better deployed.

Previous studies of the cost of induced abortion in Uganda did not consider the consequences of failed induction and the impact of abortion provider on healthcare costs [26] or did not include other aspects of health resource use such as cost of the abortion procedure, cost of treating complications, cost of transportation, and cost of patient upkeep [27]. The objective of the current study was to perform a comprehensive assessment of the economic burden of induced abortion in Uganda in terms of its costs.

## Methods

We performed a descriptive cost-of-illness study to assess the economic burden of induced abortion in Uganda. A decision tree was developed to represent the consequences of induced abortion and to estimate the cost of an average case in Uganda from a societal perspective. Data to inform the model were obtained from a primary chart abstraction study, an on-going prospective study, and the published literature. The on-going prospective study is a cohort of women enrolled following discharge after post-abortion complications, discharge after child birth, and clinic visit for contraception. It was designed to compare the women discharged following post-abortion complications with the other women groups with regard to health and economic outcomes. The total national cost of induced abortion for 2010 was estimated by multiplying the average cost by an estimate of the annual incidence of induced abortion in Uganda.

### Model structure

**Figure 1:**
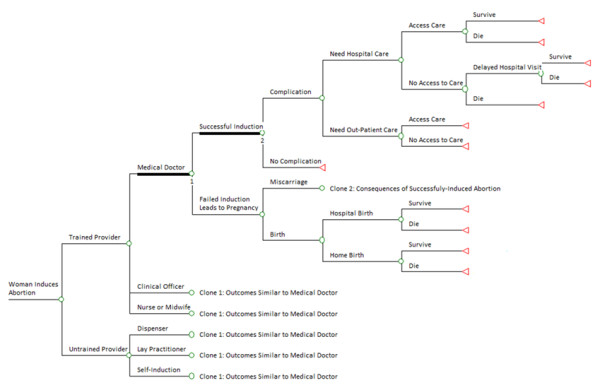
**Decision tree showing the consequences of induced abortion in Uganda**. A circle corresponds to a chance node (defined by the probability of an event occurring) and a triangle corresponds to an end node.

The decision tree showing the consequences of induced abortion is shown in Figure 1 and the probabilities used to estimate the average costs of an induced abortion case are shown in Table 1. Women who choose to abort are first divided into those who seek care from practitioners with the training to safely terminate a pregnancy and those who go to practitioners without such training. Prada et al [28]. in a study in which they interviewed health professionals, reported that the proportion of abortions induced by different providers were as follows: doctors (20%); clinical officers (17%); nurses or midwives (19%); pharmacists or dispensers in drug stores (7%); traditional healers or lay practitioners (22%); and the women themselves (15%). These estimates were used to calculate the average probability of training and abortion induction by provider assuming that doctors, clinical officers, nurses, and midwives are trained providers and dispensers, lay practitioners, traditional healers, and the women themselves are untrained providers.

**Table 1:** Average probabilities of induced abortion consequences, complications and treatment

Probability	Mean (Low, High)	Reference
Induced abortion provider		

Trained	0.56 (0.38, 0.92)	[6,28]

Untrained	0.44 (0.21, 0.52)	[6,28]

Trained provider		

Doctor	0.36 (0.29, 0.43)	[6,28]

Clinical officer	0.30 (0.24, 0.37)	[6,28]

Nurse/Midwife	0.34 (0.27, 0.41)	[6,28]

Untrained provider		

Dispenser	0.16 (0.13, 0.19)	[6,28]

Lay practitioner	0.50 (0.40, 0.60)	[6,28]

Self-induction	0.34 (0.27, 0.41)	[6,28]

Induced abortion failure		

Trained provider	0.0005 (0.0002, 0.0007)	[29-31]

Untrained provider	0.17 (0.14, 0.21)	Primary study

Complications		

Doctor	0.25 (0.17, 0.32)	[6,28]

Clinical officer	0.45 (0.33, 0.48)	[6,28]

Nurse/Midwife	0.42 (0.35, 0.43)	[6,28]

Dispenser	0.50 (0.45, 0.52)	[6,28]

Lay practitioner	0.66 (0.60, 0.68)	[6,28]

Self-induction	0.73 (0.66, 0.75)	[6,28]

Treatment of complications		

Hospital treatment	0.44 (0.35, 0.52)	[28]

Out-patient treatment	0.56 (0.45, 0.68)	[28]

Access to care	0.67 (0.51, 0.83)	[6,28]

Abortion mortality		

In-hospital	0.02 (0.01, 0.03)	[10,19]

Community	0.05 (0.03, 0.07)	Assumption

Failed abortion (Pregnancy)		

Births	0.87 (0.69, 1.00)	[6,34-36]

Miscarriage (13-22 weeks)	0.03 (0.02, 0.04)	[6,34-36]

Miscarriage (< 13 weeks)	0.10 (0.08, 0.12)	Assumption

Hospital delivery	0.39 (0.23, 0.58)	[13]

Perinatal mortality		

In-hospital	0.007 (0.005, 0.008)	[37]

Community	0.013 (0.011, 0.016)	Assumption

Women who receive abortion procedures from the different providers are further divided into those for whom induced abortion succeeds and those for whom it fails. Induced abortion rarely fails when performed by trained practitioners, and we found no studies that estimated its incidence in Uganda or similar countries, but studies in other settings have reported frequencies of 0.01% [29], 0.05% [30], and 0.07% [31]. Although these studies were performed in high-income countries, we used the estimates because no better estimates were available. The rate of abortion failure is likely higher for certain procedures or technologies which are more likely to be performed by practitioners with less training [6,27]. To estimate this probability, we calculated the incidence of second abortion attempts using data from an on-going cohort of women treated for induced abortion at Mbarara University Teaching Hospital in Uganda. According to these data, of the 47 women who received induced abortions from untrained providers, 8 needed a second attempt and 1 needed a third attempt. The initial, failed methods were: 1) herbs for 4 women, 2) an object inserted into the birth canal for 2 women, 3) crude surgical procedures for 2 women, and 4) over-the-counter medication for 1 woman. This distribution of procedures suggests that these abortion providers were untrained and this analysis uses the proportion of women needing a second or third attempt (17%) as the probability of induced abortion failure when procedures are performed by untrained providers. We assumed that when induced abortion by an untrained provider fails, women will try a trained practitioner before ultimately succeeding in terminating their pregnancy or failing and continuing with their pregnancy.

Women who have had successful induced abortions are divided in the model into those who develop complications and those who do not. The type of abortion provider has a direct influence on the probability of having abortion complications. A survey of health workers in Uganda estimated the proportion of induced abortion complications by provider [6,28]. It reported rates of abortion complications as: 25% for doctors, 42% for nurses/midwives, 45% for clinical officers, 50% for pharmacists/dispensers, 66% for traditional healers/lay practitioners, and 73% when self-induced.

Women who develop complications following induced abortion were divided into those who need out-patient care and those who need hospital care. According to Prada et al [28]. of the 109,926 estimated number of patients treated for post-abortion complications, 47,828 (43.5%) received hospital care and the rest received out-patient care. Those who need out-patient or hospital care were further divided into those who have access and those who do not have access to services. It has been reported that only 66.5% of those who need this care are able to access it depending on income and geographical location [28].

In the model, women who need and obtain hospital treatment following abortion complications both improve and are discharged alive, or they die in hospital. The in-hospital rate of abortion related mortality ranges from 1.3% [10] to 3.3% [19] in Uganda. We assumed that those who need hospital care but are unable to access it are divided into those who die at home and those who worsen and belatedly seek hospital care--a practice which has been reported in Uganda [32]. Because data were lacking for patients who do not access services, we assumed a doubling in the mortality rate in the community (compared to hospital mortality) at baseline. We assumed that women who do not access out-patient care resort to self-mediation--a practice common in Uganda [33]--and subsequently get better.

In the case of abortion failure after an attempt by a trained practitioner, the woman carries the pregnancy and faces the consequences of pregnancy. These include: a) miscarriage before 13 weeks gestation, b) miscarriage between 13 and 22 weeks which usually requires treatment, or c) birth of a child which includes preterm birth as well as term live or still birth. Miscarriages at 13-22 weeks account for 2.9% of all recognized pregnancies and live births account for 84.8% [34,35]. The rate of still births in the East Africa region is reported to be 1.9% [36]. This was added to the rate of live births to obtain the average proportion of births both live and still (86.8%). We assumed that those who miscarry between 13 and 22 weeks face health and economic consequences similar to women who suffer induced abortion. We divided women who give birth into those who give birth at home and those who give birth in health facilities. Data from Uganda suggest that 39.3% of women deliver in health facilities [13]. In a recent study in Ugandan healthcare facilities, of the 194,029 deliveries, there was a reported 1,302 deaths for an in-hospital mortality rate among women who deliver in hospital of 0.007% [37]. We assumed that the community mortality rate was at least double that at baseline.

### Estimation of the costs of induced abortion

We estimated the average cost of each outcome in the decision tree (Figure 1). The overall average cost of induced abortion is the sum of these average costs weighted by their probability of occurrence as shown in Table 1.

### Cost categories

The cost of induced abortion was considered to include the following cost categories: 1) direct medical costs, 2) direct non-medical costs, and 3) indirect (productivity) costs. Direct medical costs included personnel, medical supplies, drugs, radiology tests, laboratory tests, and patient out-of-pocket costs. Direct non-medical costs included recurrent expenditures (such as utility bills) and capital expenditures (such as expenditures on hospital infrastructure), patient transportation, and patient upkeep while seeking healthcare. Indirect costs included lost productivity while seeking abortions and getting treatment for complications as well as productivity losses from abortion morbidity while convalescing and premature abortion-related maternal mortality. The total healthcare cost is the sum of the direct medical and direct non-medical costs.

In a separate classification, the costs of induced abortion were also considered to include patient/family costs and government costs. Patient/family costs included the costs of procuring abortions, out-of-pocket costs, transportation, and upkeep while procuring abortions and seeking treatment for complications, and self-medication. Government costs included the costs of treating abortion complications and pregnancy-related costs when abortions fail, but excluded the healthcare costs associated with the procurement of abortions which are illegal in Uganda and are not provided by the national healthcare system.

The societal cost estimate is the sum of all the different kinds of costs i.e. direct medical + direct non-medical + indirect/productivity costs or patient/family costs + government costs.

### Estimation of direct medical costs of induced abortion

The cost to women of abortion services by provider were obtained from a survey of health workers (see Table 2) [28].

**Table 2:** Itemized costs (2010 $US) used in the analysis

Cost category	Base-case	Range	Reference
Abortion procedure costs			

Doctor	93.97	51.51-149.14	[28]

Clinical officer	55.68	38.12-82.26	[28]

Nurse/Midwife	42.13	28.09-62.01	[28]

Dispenser	17.05	10.03-28.09	[28]

Lay practitioner	37.12	24.08-58.16	[28]

Self-induction	11.54	8.03-18.06	[28]

Productivity	1.14	0.57-2.29	Primary study

Hospital complications			

Personnel	19.31	9.74-38.63	[26]

Supplies	11.45	10.94-11.85	[38,40] Primary study

Drugs	6.67	5.6-8.3	[38] Primary study

Diagnostic tests	14.94	14.31-15.41	[38] Primary study

Overhead and capital	10.35	8.67-12.04	[45]

Productivity	23.76	20.52 - 32.84	Primary study

Out-patient complications			

Personnel	1.01	0.51 - 2.04	[26]

Supplies	1.50	0.71 - 2.97	[38] Primary study

Drugs	1.20	1.14 - 1.36	[38] Primary study

Diagnostic tests	4.98	4.31 - 5.20	[38] Primary study

Overhead and capital	1.95	0.97 - 3.89	[45]

Productivity	1.14	0.57 - 2.29	Primary study

Other costs			

Antenatal care	10.09	5.05 - 20.18	[26]

Hospital delivery	116.63	58.32 - 233.26	[26]

Transport	2.49	1.42 - 3.03	Primary study

Upkeep	11.59	9.11 - 14.06	Primary study

Out of pocket^$^	1.48	0.74 - 2.95	Primary study

Annual productivity loss*	474.27	--	[2]

*Equivalent to GDP per capita at the real exchange rate

^$ ^Costs incurred by patients to procure inputs such as drugs and gloves that may be out of stock at a health facility

A primary chart abstraction study was performed to estimate the resource use and costs for treatment of induced abortion complications in the hospital setting. In the study, which was performed at Mbarara Hospital in Uganda, a simple random sample of 200 charts was obtained from among the patients treated for abortion complications between January 2006 and December 2008. Data on health resource use--drugs, laboratory tests, radiological tests, blood transfusions, and disposable supplies--were abstracted and used to calculate the types and amounts of resources, which were multiplied by the unit costs obtained from the price catalogue of Uganda's Joint Medical Stores [38]. Data on the unit costs of laboratory tests were obtained from a study performed in a Ugandan hospital [39]. Data on the cost of radiology tests were obtained by surveying providers in Uganda's capital Kampala. Data on the cost of a single unit of transfused blood were not available for Uganda and were obtained from a study in Malawi which is similar to Uganda [40].

The costs of healthcare personnel were based on a study in Uganda in which the personnel costs of treating abortion complications were estimated for public hospitals and missionary hospitals [26]. The unit costs of pregnancy-related care were obtained from the same study and included antenatal care as well as normal and cesarean birth [26]. These costs were adjusted for the proportion of women who attend at least 1 antenatal care visit which is 94% [41], the rate of cesarean birth which is 15.7% [42], the prevalence of common complications like post-partum hemorrhage (0.84-19.8%) [43] and eclampsia (0.53%) [44].

### Estimation of direct non-medical costs of induced abortion

The overhead and recurrent (hotel) costs of out-patient and hospital treatment of abortion complications were estimated from the World Health Organization Choosing Interventions that are Cost-Effective (WHO-CHOICE) database for Uganda [45]. Transportation and upkeep costs for patients and caregivers were estimated using data from a prospective study of women treated for post-abortion complications at Mbarara University in Uganda. This study, which is ongoing, specifically asked women how much they spent to seek healthcare services and on upkeep while they sought services.

### Estimation of indirect (productivity) costs

Productivity losses due to morbidity were estimated for both patients and caregivers using data from the prospective study by summing lost time spent in transit to hospitals (for patients and caregivers), seeking care, convalescing, and admitted to hospital (for patients and caregivers), and multiplying by wages. Wage data were obtained for formally-employed women in the same prospective study. The wage of the proportion of women who were unemployed (subsistence farmers) was valued at Uganda's gross domestic product per capita at the official exchange rate which was $474 in 2009 [2].

Productivity losses due to mortality were estimated using the human capital approach [46] valuing lost productivity based on GDP per capita for wage and the life expectancy for Ugandan women at age 28, the average age of women receiving treatment for induced abortion complications, obtained from World Health Organization life tables for Ugandan women [47]. Future costs were discounted at 3% per year.

The unit costs used in the analysis are summarized in Table 2.

### Analysis

All costs were converted into United States dollars ($US) using the Bank of Uganda official exchange rate on 1st June 2010 [48] and were adjusted to the year 2010 using Uganda's Consumer Price Index for health [49]. To take into account the potentially large amount of uncertainty in many of the parameter estimates, distributions were defined for each uncertain parameter estimate using the mean and the standard error estimated based on the assumption that all the ranges represented a 95% confidence interval (equal to four times the standard error) [50]. Beta distributions were used for probabilities and normal distributions for costs. The model was run 10,000 times and on each occasion, a new set of estimates was randomly selected according to their distribution using Monte Carlo simulation. This provided an outcome distribution of the cost of an average case of induced abortion and allowed the reporting of a mean and a 95% credibility range (95% CRs) around the estimate. Univariate uncertainty analysis was also performed to determine which variables had the greatest influence on costs. The uncertainty analyses were performed using TreeAge Pro.

## Results

### Cost per average case of abortion

The average costs of induced abortion are shown in Table 3. The average societal cost per induced abortion (95% credibility range) was $177 ($140-$223). The average direct medical cost was $65 ($49-$86) and the average direct non-medical cost was $19 ($16-$23). The average indirect (productivity) cost was $92 ($57-$139). Patients incurred an average of $62 ($46-$183), 73% of the healthcare costs of induced abortion while government incurred an average of $14 ($10-$20), 17% of the healthcare costs of induced abortion.

**Table 3:** Average costs ($ US, Year 2010 values) of induced abortion in Uganda by the different cost categories

Cost	Mean	SD	95% CR	Minimum	Maximum
Direct Medical	65.3	9.4	49.3 - 86.4	39.3	113.1

Direct Non-Medical	19.4	1.9	15.9 - 23.3	13.4	28.5

Indirect/Productivity	92.4	21.2	57.2 - 138.7	37.5	198.0

Government	14.0	2.7	9.7 - 20.2	6.5	31.2

Patient	61.7	9.4	46.2 - 83.2	31.1	110.0

Societal	177.4	21.5	139.6 - 223.3	114.6	275.4

SD - Standard Deviation; CR - Credibility Range

### National estimates

The annual incidence of induced abortion in Uganda is 362,000 cases. Therefore the national annual expenditure on induced abortion is projected to be $23.6 million in direct medical costs, $7.0 million in direct non-medical costs, $33.5 million in indirect/productivity costs, $5.1 million in costs to the government, $22.3 million in costs to patients, and $64.2 million in societal costs.

### Sensitivity analysis

**Figure 2:**
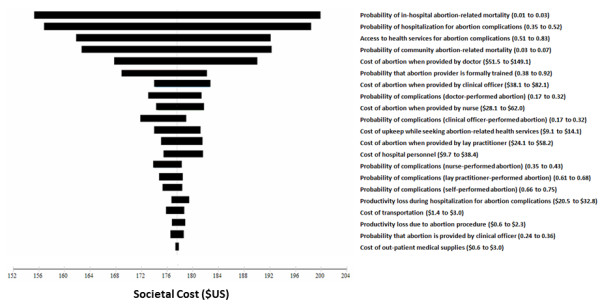
**Tornado diagram of univariate sensitivity analysis**. The 20 most influential variables are shown.

Univariate sensitivity analysis (Figure 2) showed that the societal cost of an average induced abortion was most sensitive to the uncertainty associated with the probability of in-hospital abortion-related mortality, the probability of hospitalization for abortion complications, access to health services for abortion complications, and community abortion-related mortality.

## Discussion

Using a decision tree and data from multiple sources, we found that the average induced abortion in Uganda was associated with $177 in societal costs. This is over four times the per capita expenditure on health care in Uganda which is $44 [25]. Given the 362,000 incident induced abortions annually [15], this amounts to $64 million in annual spending on induced abortions which is 4% of the approximately $1.5 billion in annual national health expenditure in Uganda [2,25]. The bulk of the societal costs (52%) were productivity costs and the remaining 48% were healthcare costs. The government, which is responsible for providing healthcare in Uganda, incurred only 17% of the total healthcare costs with the bulk of the total healthcare costs (83%) incurred by patients/families.

The annual abortion expenditure in Uganda is substantial and is a testament to the economic impact of abortion in countries where it is illegal (and likely to be unsafe), which has been previously described [51]. The proportion of healthcare costs (17%) that are incurred by the government, which is, in theory, responsible for providing healthcare to all citizens, is surprising. This may be the reason for the policy maker apathy that characterizes efforts to reduce unsafe abortions in Uganda; the government faces only a small fraction of the costs and the problem remains invisible to government policy makers.

The largest contributor to the average societal cost of induced abortion was productivity costs (52%) but the healthcare component, of which direct medical costs were the largest, was also substantial (48%). The key driver of productivity costs is abortion-related mortality. The loss of young mothers from abortion exerts a substantial burden to society. The largest part of the healthcare costs can be attributed to the treatment of the complications of unsafe abortions. The majority of trained and untrained providers choose surgical techniques--such as evacuation and manual vacuum aspiration--to terminate pregnancies but not misoprostol combined with mifepristone which is the safest form of medical abortion [52]. However, because abortion is illegal, the drug cannot be openly imported or sold for the abortion indication. A limited amount is probably used off-label after importation for post-partum hemorrhage and incomplete abortion, but this is likely not enough to improve safety and reduce costs.

The average healthcare cost (direct medical and direct non-medical) of induced abortion was $85. This estimate included the cost of abortion procedures as well as the treatment of post-abortion complications. Levin et al [26]. estimated a cost of treatment of abortion complications in 2003 of $35 in public hospitals and $58 in mission hospitals in Uganda. And another study by Johnstone et al [53]. used data mostly from Uganda to study the costs to health systems of unsafe abortions under four service delivery scenarios. They estimated mean per case cost of abortion care of $45 in settings, such as Uganda, that place heavy restrictions on elective abortion and a conventional approach to post-abortion care. Our higher estimate can be at least partly explained by inflation, our inclusion of the cost of abortion procedures, costs of pregnancies when abortion fails, and other direct non-medical costs such as patient transport and upkeep.

Vlassoff et al [54]. estimated a cost for post-abortion care in Africa of $83 in 2006. While this estimate is close to ours, it represents an estimate from three countries including Uganda and the Ugandan estimate was the lowest ($10 in Uganda, $50 in Ghana, and $112 in Nigeria) [54]. Another analysis by Shearer et al [55]. estimated a higher cost per case of treating post-abortion complications of $392 but their analysis also represents pooled unit costs from many countries which likely drove the cost estimate upwards.

One limitation of this study was the use of estimates of the proportion of abortions induced by different providers, the rate of complications, and costs of abortion procedures obtained from a survey of health workers [28] as opposed to a prospective observational study. While these data may not be the most accurate, they are the best estimates available. And since ethical justification for the performance of more accurate studies of abortion in the Ugandan setting given legal proscriptions would be difficult, innovative research studies that would improve estimates without crossing ethical and legal barriers are needed. The inevitable uncertainty in these estimates was accounted for by using Monte Carlo simulation.

Another limitation of the study is that we did not include intangible societal costs of abortion such as the non-labor force value of women in taking care of children and the potential multi-generational effect of orphan hood. While these effects have been demonstrated previously, there is a need for Uganda-specific research to better inform policies aimed at reducing the impact of unsafe abortion. Additional data limitations to our study include the use of some resource use estimates from an on-going cohort study before completion of the protocol due to time and resource limitations, the failure to distinguish between the costs of legal and illegal induced abortions in the analysis as well as use of data on induced abortion failure from developed countries due to a lack of estimates from Uganda or similar countries. As mentioned above, the inherent uncertainty in these estimates was addressed by using Monte Carlo simulation.

The results of the sensitivity analysis showed that the estimate of costs of induced abortion in Uganda were most sensitive to the uncertainty surrounding in-hospital mortality from abortion complications and the rate of hospitalization for complications. The sensitivity of the societal cost to a relatively narrow range (1-3%) of in-hospital mortality, as well as the fact that productivity losses contribute over half (52%) of the total societal cost, point to the substantial economic burden of abortion-related mortality. Data on the rate of abortion complications were derived from surveys of health workers as opposed to prospective studies. It will require innovative research methods to improve on the quality of these estimates in the prevailing legal environment or a change in the abortion law which seems like a remote possibility at the current time.

## Conclusion

The substantial cost associated with the average induced abortion in Uganda, reinforces the case made by other researchers--that efforts to reduce unsafe abortions by increasing contraceptive coverage or providing safe, legal abortions, are critical. Such efforts would contribute to achieving Millennium Development Goal number 5 (improve maternal health) and are in line with the United States Global Health Initiative which places an emphasis on improving the health of women. The study may also be used as a basis for estimation of the potential economic gains of policies that increase contraceptive coverage or provide safe, legal abortions.

## Competing interests

The authors declare that they have no competing interests.

## Authors' contributions

JBB conceived of the study and participated in the design of the study, collecting data, performing the analysis and drafting the manuscript. AS participated in designing the study and revising the manuscript. DLV participated in designing the study and revising the manuscript. JSG participated in designing the study and revising the manuscript. JN participated in collecting data and revising the manuscript. PM participated in collecting data and revising the manuscript. LPG participated in designing the study and revising the manuscript. All authors have read and approved the final manuscript.

## Pre-publication history

The pre-publication history for this paper can be accessed here:

http://www.biomedcentral.com/1471-2458/11/904/prepub

## References

[B1] Republic of Uganda, Penal Code Act (CAP 106), Revised Edition 1984

[B2] CIA-World Factbook--Uganda https://www.cia.gov/library/publications/the-world-factbook/geos/ug.html#Econ Accessed 13th July 2010

[B3] Uganda Catholic Bishops' Conference, Open Letter to the government and people of Uganda, 2006 http://www.catholicculture.org/library/view.cfm?recnum=6792&longdesc accessed April 16th 2008

[B4] Rice A Evangelicals vs. Muslims in Africa; enemy's enemy, New Republic 2004

[B5] Namagembe I Study of the knowledge, practices and attitudes of men from Kampala institutions towards illegally induced abortions, study presented at the 1st meeting of East, Central and Southern Africa Association of Obstetrical and Gynecological Societies, Kampala, Uganda 1997 5 9

[B6] Singh S Unintended pregnancy and induced abortion in Uganda: causes and consequences 2006 New York: Guttmacher Institute

[B7] Uganda Bureau of Statistics (UBOS) and Macro International Inc. 2007. Uganda Demographic and Health Survey 2006. Calverton, Maryland USA: UBOS and Macro International Inc

[B8] Mirembe F A situational analysis of induced abortions in Uganda Afr J Fertil Sexual Reprod Health 1996 1 1 79 80 12159505

[B9] Bankole A Singh S Haas T Characteristics of women who obtain induced abortion: a worldwide review Int Fam Plann Perspec 1999 25 2 68 77 10.2307/2991944

[B10] Kinoti SN Gaffikin L Benson J How research can affect policy and programme advocacy: example from a three-country study on abortion complications in sub-Saharan Africa East Afr Med J 2004 81 2 63 70 15125088 10.4314/eamj.v81i2.9127

[B11] Kaye DK Mirembe FM Bantebya G Johansson A Ekstrom AM Domestic violence as risk factor for unwanted pregnancy and induced abortion in Mulago Hospital, Kampala, Uganda Trop Med Int Health 2006 11 1 90 101 10.1111/j.1365-3156.2005.01531.x 16398760

[B12] Okonofua F Abortion and maternal mortality in the developing world J Obstet Gynaecol Can 2006 28 11 974 979 17169222 10.1016/S1701-2163(16)32307-6

[B13] Uganda Bureau of Statistics and ORC Macro, Uganda Demographic and Health Survey 2000-2001, Kampala, Uganda 2001 Uganda Bureau of Statistics; and Calverton, MD, USA: ORC Macro

[B14] AbouZhar C Wardlaw T Maternal Mortality in 2000: Estimates of Developed by WHO, UNICEF and UNFPA 2003 Geneva: World Health Organization (WHO)

[B15] Singh S Prada E Mirembe F Kiggundu C The incidence of induced abortion in Uganda Int Fam Plan Perspect 2005 31 4 183 191 10.1363/3118305 16439346

[B16] Vlassoff M Benefits of meeting the contraceptive needs of Ugandan women Brief 2009 4 New York: Guttmacher Institute 19938236

[B17] Division of Reproductive Health Unsafe Abortion Global and Regional Estimates of the Incidence of Unsafe Abortion and Associated Mortality in 2000 2004 fourth Geneva: WHO

[B18] Benson J Nicholson LA Gaffikin L Kinoti SN Complications of unsafe abortion in sub-Saharan Africa: a review Health Policy Plan 1996 11 2 117 131 10.1093/heapol/11.2.117 10158454

[B19] Bazira ER Induced abortion at Mulago Hospital Kampala, 1983-1987: a case for contraception and abortion laws' reform Trop Heal 1992 11 1 13 16 12319272

[B20] Uganda Ministry of Health and UNICEF Status of emergency obstetric care (EmOC) in Uganda: a national needs assessment of EmOC process indicators 2003 New York: UNICEF

[B21] Africa initiatives Addressing obstetric and neonatal complications in Africa from community and facility perspectives, descriptive reports from Ghana, Malawi and Uganda, Arlington 1998 VA, USA: John SnowInc.

[B22] Okonofua FE Onwudiegwu U Odunsi OA Illegal induced abortion: a study of 74 cases in Ile-Ife, Nigeria Trop Doct 1992 22 2 75 78 1604719 10.1177/004947559202200209

[B23] Madebo T Tsadic TG A six month prospective study on different aspects of abortion Ethiop Med J 1993 31 3 165 172 8404881

[B24] Goyaux N Alihonou E Diadhiou F Leke R Thonneau PF Complications of induced abortion and miscarriage in three African countries: a hospital-based study among WHO collaborating centers Acta Obstet Gynecol Scand 2001 80 6 568 573 10.1080/j.1600-0412.2001.080006568.x 11380296

[B25] World Health Statistics 2011. World Health Organization http://www.who.int/whosis/whostat/2011/en/index.html

[B26] Levin A Dmytraczenko T McEuen M Ssengooba F Mangani R Van Dyck G Costs of maternal health care services in three anglophone African countries Int J Health Plan Manage 2003 18 1 3 22 10.1002/hpm.690 12683270

[B27] Jagwe-Wadda G Abortion morbidity in Uganda: evidence from two communities, Occasional Report 2006 26 New York: Guttmacher Institute

[B28] Prada E Abortion and postabortion care in Uganda: a report from health care professionals and health facilities, Occasional Report 17 New York: The Alan Guttmacher Institute

[B29] Bozorgi N Statistical analysis of first-trimester pregnancy termination in an ambulatory surgical center Am J Obstet Gynecol 1977 127 763 848529 10.1016/0002-9378(77)90254-x

[B30] Wulff GJL Freiman SM Elective abortion: complications seen in a free-standing clinic Clin Obstet Gynecol 1974 120 802 840464

[B31] Fielding WL Lee SY Friedman EA Continued pregnancy after failed first trimester abortion Obstet Gynecol 1978 52 1 56 58 683631

[B32] Kallander K Hildenwall H Waiswa P Galiwango E Peterson S Pariyo G Delayed care seeking for fatal pneumonia in children aged under five years in Uganda: a case-series study Bull World Health Organ 2008 86 5 332 338 10.2471/BLT.07.049353 18545734 PMC2647445

[B33] Anyama N Adome RO Community pharmaceutical care: an 8-month critical review of two pharmacies in Kampala Afr Health Sci 2003 3 2 87 93 12913800 PMC2141595

[B34] Bongaarts J Potter RG Fertility, Biology and Behavior: an Analysis of the Proximate Determinants 1983 New York: Academic Press

[B35] Harlap S Shiono PH Ramcharan S Hook EB, Porter I A life table of spontaneous abortions and the effects of age, parity and other variables Human Embryonic and Fetal Death 1980 New York: Academic Press

[B36] Stanton C Lawn JE Rahman H Wilczynska-Ketende K Hill K Stillbirth rates: delivering estimates in 190 countries Lancet 2006 367 9521 1487 1494 10.1016/S0140-6736(06)68586-3 16679161

[B37] Mbonye AK Mutabazi MG Asimwe JB Sentumbwe O Kabarangira J Nanda G Orinda V Declining maternal mortality ratio in Uganda: priority interventions to achieve the Millennium Development Goal Int J Gynaecol Obstet 2007 98 3 285 290 10.1016/j.ijgo.2007.05.019 17617415

[B38] Joint Medical Stores (JMS). Catalogue and Price Indicator August 2009 http://www.jms.co.ug Accessed on July 20th 2010

[B39] Ninci A Ocakacon R How much do lab tests cost? Analysis of Lacor Hospital laboratory services Health Policy Dev 2 2 44 150

[B40] Lara AM Kandulu J Chisuwo L Kashoti A Mundy C Bates I Laboratory costs of a hospital-based blood transfusion service in Malawi J Clin Pathol 2007 60 10 1117 1120 10.1136/jcp.2006.042309 17412875 PMC2014856

[B41] UNICEF Country Statistics for Uganda Accessed at [http://www.unicef.org/infobycountry/stats_popup8.html on July 16th 2010]

[B42] Shah A Fawole B M'Imunya JM Amokrane F Nafiou I Wolomby JJ Mugerwa K Neves I Nguti R Kublickas M Cesarean delivery outcomes from the WHO global survey on maternal and perinatal health in Africa Int J Gynaecol Obstet 2009 107 3 191 197 10.1016/j.ijgo.2009.08.013 19782977

[B43] Lalonde A Daviss BA Acosta A Herschderfer K Postpartum hemorrhage today: ICM/FIGO initiative 2004-2006 Int J Gynecol Obstet 2006 94 3 243 253 10.1016/j.ijgo.2006.04.016 16842791

[B44] Kidanto HL Mogren I Massawe SN Lindmark G Nystrom L Criteria-based audit on management of eclampsia patients at a tertiary hospital in Dar es Salaam, Tanzania BMC Pregnancy Childbirth 2009 9 13 10.1186/1471-2393-9-13 19323846 PMC2670267

[B45] Choosing Interventions that are Cost-Effective. Estimates of Unit Costs of Patient Services in Uganda Accessed at [http://www.who.int/choice/country/uga/cost/en/index.html on July 20th 2010]

[B46] Goeree R O'Brien BJ Blackhouse G Agro K Goering P The valuation of productivity costs due to premature mortality: a comparison of the human-capital and friction-cost methods for schizophrenia Can J Psychiatry 1999 44 5 455 463 10389606 10.1177/070674379904400505

[B47] World Health Organization, Global Health Observatory. Life Tables for Countries: Uganda Accessed at [http://apps.who.int/ghodata/?vid = 720 on July 16th 2010]

[B48] Bank of Uganda Accessed at [http://www.bou.or.ug/bouwebsite/opencms/bou/home.html on July 20th 2010]

[B49] Uganda Bureau of Statistics. Consumer Price Index June 2010 Accessed at [http://www.ubos.org/onlinefiles/uploads/ubos/cpi/junecpi2010/June%202010%20CPI_Publication.pdf on July 20th 2010]

[B50] Briggs AH Handling uncertainty in cost-effectiveness models Pharmacoeconomics 2000 17 5 479 500 10.2165/00019053-200017050-00006 10977389

[B51] Vlassoff Michael Shearer Jessica Walker Damian Lucas Henry Economic impact of unsafe abortion-related morbidity and mortality: evidence and estimation challenges. IDS Research Report 59 Institute of Development Studies 2008

[B52] Kawonga M Blanchard K Cooper D Cullingworth L Dickson K Harrison T von Mollendorf C Winikoff B Integrating medical abortion into safe abortion services: experience from three pilot sites in South Africa J Fam Plann Reprod Health Care 2008 34 3 159 164 10.1783/147118908784734846 18577314

[B53] Johnston HB Gallo MF Benson J Reducing the costs to health systems of unsafe abortion: a comparison of four strategies J Fam Plann Reprod Health Care 2007 33 4 250 257 10.1783/147118907782101751 17925105

[B54] Vlassoff M Walker D Shearer J Newlands D Singh S Estimates of health care system costs of unsafe abortion in Africa and Latin America Int Perspect Sex Reprod Health 2009 35 3 114 121 10.1363/3511409 19805016

[B55] Shearer JC Walker DG Vlassoff M Costs of post-abortion care in low- and middle-income countries Int J Gynaecol Obstet 108 2 165 169 10.1016/j.ijgo.2009.08.037 20035938

